# Efficacy and safety of sacubitril/valsartan in the treatment of heart failure: protocol for a systematic review incorporating unpublished clinical study reports

**DOI:** 10.12688/hrbopenres.12951.2

**Published:** 2021-04-01

**Authors:** David Byrne, Tom Fahey, Frank Moriarty

**Affiliations:** 1 Department of General Practice and HRB Centre for Primary Care Research, Royal College of Surgeons in Ireland, Dublin 2, Ireland

**Keywords:** Systematic Review, Clinical Study Reports, Sacubitril/valsartan, Heart Failure

## Abstract

**Background:** Sacubitril/valsartan is a first-in-class angiotensin-receptor neprilysin inhibitor used to treat heart failure. The evidence for this novel medication is largely based on one pivotal phase III trial which was stopped early due to significant clinical benefits being shown. However potential limitations in trial design have been highlighted in recent literature, necessitating a thorough review of all evidence for sacubitril/valsartan.

**Methods:** This review will be conducted using the PRISMA reporting guidelines. Relevant randomised controlled trials (RCTs) for sacubitril/valsartan will be systematically searched for in Medline (PubMed), Embase, Cochrane library, Google Scholar, Web of Science, Toxline and Scopus. Clinical trials registries will be searched, as will eight grey literature databases. In addition, unpublished clinical study reports (CSRs) of relevant trials will be requested from the European Medicines Agency (EMA) and the Clinical Study Data Request database. Studies will be included if they involve randomising adult patients with heart failure to either sacubitril/valsartan or usual care, with either an active comparator or placebo as a control. Heart failure of any subtype or NYHA class will be included. All relevant clinical and safety outcomes will be reviewed, particularly hospitalisation due to heart failure and cardiovascular mortality. Two reviewers will assess eligibility of selected studies for inclusion. Data extraction will be performed separately for trial publications, clinical trial registries and for CSRs using a piloted form. Methodological quality of included trials from published sources will be assessed separately using the Cochrane Risk of Bias tool (RoB 2). Narrative synthesis of included studies will be conducted and, if appropriate, meta-analysis for clinical efficacy and safety outcomes.

**Discussion:** This review will collate all available RCT data on sacubitril/valsartan including published and unpublished sources in order to obtain a more complete picture of the evidence base for sacubitril/valsartan.

**Registration: **This protocol is registered on PROSPERO (reference CRD42020162031).

## Introduction

### Rationale

Heart failure is a common chronic disease with an estimated prevalence of 1–2% in developed countries making it more common than most cancers, with the majority affected being over the age of 70 ^1, 2^. It is a significant public health burden with high morbidity and mortality and is one of the most common reasons for emergency medical admissions ^1^. Sacubitril/valsartan (also known as LCZ696 or Entresto®), is a first in class angiotensin-receptor neprilysin inhibitor used in the treatment of heart failure with reduced ejection fraction (HFrEF) ^3, 4^.

The enzyme neprilysin acts by degrading various vasoactive substances including natriuretic peptides which results in enhanced diuresis, natriuresis and vasodilatation ^3, 5^. However neprilysin also increases angiotensin II, leading to sodium retention and vasoconstriction ^5^. Combined simultaneous inhibition of the RAAS system, using an angiotensin II receptor blocker, for example valsartan, is therefore required to produce potential benefits in heart failure ^5, 6^

Sacubitril/valsartan was approved by both the European Medicines Agency (EMA) and Food and Drug Administration (FDA) in 2015 ^4^. It was recommended for use in HFrEF by the National Institute for Health and Clinical Excellence (NICE) in 2016 ^7^. It is now recommended in the American Heart Association (AHA) Guideline for the Management of Heart Failure as a replacement for ACE inhibitors for patients with New York Heart Association (NYHA) class II or III HFrEF who are persistently symptomatic despite optimal medical management ^8^. It is also a recent addition to the European Society of Cardiology (ESC) and NICE guidelines for the management of heart failure, in those with NYHA class II-IV HFrEF ^2, 9^. It was approved by the National Centre for Pharmacoeconomics (NCPE) for reimbursement on the Irish state drug schemes in 2016 for an estimated potential 18,500 patients with HFrEF ^10^

Regulatory approval for sacubitril/valsartan was mostly based on one pivotal phase III trial, PARADIGM-HF, involving 8,422 participants ^3^. For the primary outcome, a composite endpoint was used which was defined as cardiovascular death or hospitalisation due to heart failure ^3^. As reported on Clinicaltrials.gov, the original planned primary outcome was time to first occurrence of this composite endpoint. This was changed during the course of the trial with the eventual primary outcome being the total number of participants with the first occurrence of the composite endpoint ^3^. The trial ceased early after a median follow up of 27 months due to an apparent significant benefit with sacubitril/valsartan being shown, with the planned follow up being 51 months ^3^. This study had used a single-blind run-in phase during which time all patients received the control drug, followed by a second single-blind run-in phase with sacubitril/valsartan with an overall attrition rate of almost 20% ^11^. While this method may reduce treatment discontinuation and improve internal validity, it may reduce the external validity and generalisability of a trial ^12^. Non-equivalent dosing between sacubitril/valsartan and the comparator drug, enalapril, in the two arms of this trial and also NYHA classification heterogeneity have previously been discussed ^11, 12^, all of which may potentially impact the reliability of the evidence for this drug.

An earlier randomised, double blind phase 2 trial of sacubitril/valsartan compared with valsartan, the PARAMOUNT-HF study, used surrogate endpoints, namely a change in NT-proBNP (N-terminal-pro hormone B-type natriuretic peptide) as primary endpoint and specific echocardiographic changes as its main secondary endpoint ^13^. This study showed no significant differences in NYHA class or patient-related quality of life scores using the Kansas City Cardiomyopathy Questionnaire (KCCQ) between intervention and control groups at 12 weeks. A significant improvement in NYHA class at 36 weeks was reported.

In order to obtain a more complete picture of the evidence base for sacubitril/valsartan, we aim to complete a thorough systematic review and meta-analysis of all available randomised controlled trials (RCTs) for this novel medication, to include both published and unpublished sources. The importance of including unpublished evidence in decision making for medications has been illustrated in a number of studies and systematic reviews ^14^. Documents used to obtain regulatory approval for medications, including Clinical Study Reports (CSRs), provide a rich and authoritative description of RCTs, providing details on study design, methods, and results which may be absent or inaccurate in published papers ^15, 16^.

Systematic reviews of antiviral medications for influenza incorporating unpublished CSRs refuted a previously demonstrated clinical benefit ^17, 18^ which would likely have changed clinical and healthcare resources decisions ^19^. Even when trials are published, important elements of study design, methods and results may be under- or misreported, hampering risk of bias assessment, critical appraisal, and assessing the strength of evidence using the GRADE approach for instance ^20^. Relying only on published evidence may distort the benefit/risk ratio and result in policy-makers, patients, and clinicians making sub-optimal decisions ^21^.

### Research question

In patients with heart failure taking sacubitril/valsartan, are clinical outcomes improved compared with those on standard therapy when all RCT evidence, including unpublished trial evidence, is considered?

### Aim and objective

***Aim.*** The aim of this systematic review is to explore the evidence for the efficacy and safety of sacubitril/valsartan in patients with heart failure.

***Objective.*** The objective of this systematic review is to synthesise all available RCT evidence on the efficacy and safety of sacubitril/valsartan, compared to usual care or placebo, in patients with heart failure and to determine the estimated clinical benefits and harms.

## Methods

### Eligibility criteria

RCTs will be eligible if they investigate the clinical efficacy and safety of sacubitril/valsartan in patients with chronic heart failure and which fulfil the criteria.

***Participants.*** We will include all RCTs involving adults aged 18 or over with NYHA class I-IV heart failure. The pivotal study of sacubitril/valsartan, PARADIGM-HF included those with HFrEF of 35% or less ^3^. However, the use of this medication in heart failure with preserved ejection fraction (HFpEF) has also been investigated and a significant reduction in total hospitalisation or cardiovascular death was not shown ^22^. For the purposes of this review we will include all subtypes of heart failure.

***Interventions and comparators.*** RCTs will be included if they randomise patients to use sacubitril/valsartan or to usual care with either an active comparator or placebo as a control.

***Outcomes***

The outcomes of interest for analysis will include:

**Efficacy outcomes**

- Death from cardiovascular causes
- Hospitalisation due to heart failure
- Non-fatal cardiovascular events
- All-cause mortality
- Change in relevant patient-reported quality of life scores (KCCQ and EuroQol/EQ-5D)
- Change in NYHA functional class
- Days alive outside of hospital
- Time to treatment failure
- Health resource utilisation (Emergency Department visits and Intensive Care Unit stays)
- Change in NT-proBNP

**Safety outcomes**

- Impairment in renal function
- Change in estimated glomerular filtration rate (eGFR)
- New onset atrial fibrillation
- Non-fatal cardiovascular events
- Symptomatic hypotension
- Falls
- Angioedema
- All other relevant clinical and safety outcomes will be considered, including those from published core outcome sets for heart failure ^23^.

For outcomes reported as time-to-event/time-to first occurrence, data on event rate, total count and proportion of participants with any occurrence will also be analysed where available.

### Study design

The systematic review and meta-analysis will be conducted using the Preferred Reporting Items for Systematic reviews and Meta-Analysis Protocols (PRISMA) reporting guidelines ^24^. This protocol is structured using the PRISMA-P guidelines ^25^.

### Information sources and search method

RCTs will be searched for via the most relevant traditional medical databases for systematic reviewing including Medline (PubMed), Embase, Web of Science and Google Scholar ^26^ as well as those specific to this topic including Cochrane Central Register of Controlled Trials (CENTRAL) and Toxline. Selected clinical trials registries namely clinicaltrials.gov, EU Clinical Trials Register and WHO International Clinical Trials Registry Platform (ICTRP), will also be searched for eligible trials. Grey literature databases will be searched to identify trials in, for example, conference abstracts and also regulatory approval documents from the FDA and EMA. Citation searching will be carried out using Scopus and Web of Science, as well as reference list searching for all included studies. A full list of databases to be searched is included in *Extended data* ^27^.

Databases will be searched from inception using the appropriate search strategy and there will be no language restrictions. All trials with available results will be included. Duplicate records will be removed, using clinical trial registry number or other approaches to identify matching studies.

In addition to publications and clinical trial registry entries, for identified trials, relevant unpublished CSRs for sacubitril/valsartan will be requested from the European Medicines Agency (EMA) and the Clinical Study Data Request (CSDR) database. Relevant CSRs will be identified by examining the EMA’s regulatory approval documents for sacubitril/valsartan, namely European Public Assessment Reports (EPARs) and requesting CSRs for all clinical RCTs cited in this approval process.

### Search strategy

The relevant search strategies for both for PubMed and Embase are included in *Extended data* ^27^, which will be finalised with the assistance of an information specialist. Both search strategies use a recommended validated filter from the Cochrane Handbook for identifying randomised trials. To ensure that as many trials as possible are included for this novel medication, we will apply the sensitivity-maximising version of the Cochrane RCT filter (2008 revision) for PubMed and relevant adaptation for Embase ^28^. For all other databases, RCTs will be searched for using medication names ‘sacubitril/valsartan’ or ‘Entresto’ and search terms related to study design e.g. ‘randomised controlled trial’.

### Study records

***Data management.*** Relevant search results will be exported and stored in Endnote X8 reference manager and duplicates will be removed. The Covidence systematic review management system will be used for title and abstract screening and full text reviewing. The phases of the systematic review will be recorded using a PRISMA flow diagram. Review Manager Software (RevMan) version 5.3 will be used for further analysis including meta-analysis, if appropriate.

The length of CSR documents are generally several hundreds, up to several thousands of pages in length. The use of CSRs in this review will add a significant volume of data to be managed. The EMA have developed a guideline on the suggested structure and content of CSRs in conjunction with the International Council for Harmonisation (ICH) of Technical Requirements for Registration of Pharmaceuticals for Human Use ^29^. For each included study in this review, relevant sections of each CSR document will be indexed using file name and page number as per this ICH standard. This will facilitate more systematic searching of the data within these documents.

***Selection process.*** Two reviewers will independently screen titles and abstracts of identified studies to include those that are relevant. Remaining studies will then be assessed for eligibility. The two reviewers will independently read the full text records to determine if the studies are eligible for inclusion, and disagreements will be managed by consensus.

***Data collection process.*** Data extraction will be performed separately for trial publications and trial registries, as well as for CSRs. This will be carried out using a standardised form which will be iteratively developed and piloted and will be recorded using a Microsoft Excel database. For any missing data or for data presented in a form that is not suitable for meta-analysis, corresponding authors will be contacted by email to request such data, up to a maximum of three attempts.

***Data items.*** Data to be extracted from included trials will include information on study design, methodological characteristics (for quality assessment), pre-specified trial outcomes, trial participant baseline descriptive characteristics, information on the intervention, clinical efficacy and safety outcomes and results. For outcomes reported as time-to event, data on the relevant event rate and total count will be collected or, if not reported, will be requested.

### Risk of bias in individual studies

Methodological quality of included trials from published sources will be assessed separately using the recently updated Cochrane Risk of Bias tool version 2 (RoB 2), assessing bias across five domains, namely the randomisation process, deviations from intended interventions, missing outcome data, outcome measurement and selection of the reported result ^30^. This will be conducted independently by two reviewers with any discrepancies or disagreements resolved with the assistance of a third reviewer. 

### Meta-biases

Specific bias of interest will include allocation bias, attrition bias (RoB 2), selective outcome reporting and bias related to trial funding. Publication bias will be assessed using funnel plots.

### Data synthesis

A narrative synthesis of included studies will be conducted. If appropriate, based on study homogeneity, meta-analysis will be undertaken where possible for included efficacy and safety outcomes using appropriate random-effects regression models (treatment effect varying across studies). In terms of treatment effect measures, relative risk (RR) with 95% confidence interval (CI) will be used for dichotomous data (e.g. cardiovascular mortality) and mean difference or standardised mean difference will be used for continuous data. For event rate data (e.g. numbers of hospitalisations) incidence rate ratio will be used and for time-to-event data (e.g. time to first hospitalisation) hazard ratio will be used.

Data will be extracted separately from each source, namely trial publication, trial registry and CSR. Both quantitative and qualitative data will be extracted including clinical efficacy and safety outcomes as well as, where relevant, details of trial characteristics and design. Meta-analyses will be conducted using all available evidence, with a sensitivity analysis using only published sources. In cases of outcome data discrepancy between trial publication, trial registry entry or CSR, then the CSR data will be used in preference as the presumed most reliable source, followed by trial registry data. Sensitivity analysis will then also be conducted for each outcome to meta-analyse data from each source separately in order to assess the impact on the effect estimate

Heterogeneity in outcomes due to study characteristics will be evaluated using Higgins I ^2^ test initially (I ^2 ^>50%), as well as meta-regression if sufficient studies are identified. For composite outcomes, where possible, data for each outcome will be requested, recorded and analysed separately.

Analysis will be performed using pooled data of all heart failure subtypes. Where estimates of efficacy and safety are available for individual heart failure subtypes and where consistent definitions of ejection fraction are used across studies, analysis will be performed separately for any subtypes e.g. preserved ejection fraction and reduced ejection fraction.

### Confidence in cumulative evidence

The quality of all evidence from the studies will be assessed using the Grading of Recommendations, Assessment, Development and Evaluation (GRADE) methodology ^31^, in addition to adherence to CONSORT standardised reporting guidelines.

## Ethical considerations

For this study no primary identifiable patient data will be collected, obtained or analysed. All data will be from secondary sources and will be anonymised (e.g. data from published medical journals, trials from unpublished sources). As such, ethical approval will not be sought.

## Protocol amendments

In the event of any amendments to this protocol, the description including the rationale and date of such a change will be documented.

## Data availability

### Underlying data

No underlying data are associated with this article.

### Extended data

Open Science Framework: Efficacy and safety of sacubitril/valsartan in the treatment of heart failure: protocol for a systematic review incorporating unpublished clinical study reports. https://doi.org/10.17605/OSF.IO/MBJRG ^27^.

This project contains the following extended data:

- SacubitrilValsartan Embase Search Strategy.pdf (search strategy for Embase).
- SacubitrilValsartan Pubmed Search Strategy.pdf (search strategy for PubMed).
- SacubitrilValsartan SR Search Databases.pdf (list of databases to be searched).

### Reporting guidelines

Open Science Framework: PRISMA-P checklist for ‘Efficacy and safety of sacubitril/valsartan in the treatment of heart failure: protocol for a systematic review incorporating unpublished clinical study reports’. https://doi.org/10.17605/OSF.IO/MBJRG ^27^.

Extended data and completed reporting guidelines checklist are available under the terms of the Creative Commons Attribution 4.0 International license (CC-BY 4.0).
