# Supplementary material for: Efficacy and safety of sacubitril/valsartan in the treatment of heart failure: protocol for a systematic review incorporating unpublished clinical study reports
Source: HRB Open Res. 2021 Apr 1;3:5. Originally published 2020 Feb 10. [Version 2] doi: 10.12688/hrbopenres.12951.2 (PMC7233178; doi:10.12688/hrbopenres.12951.2)
Supplement: Peer review history

# Peer review: Reviewer response for version 1

**DOI:** 10.21956/hrbopenres.14033.r27364

**Published:** 2020-05-15

**Authors:** Andrea Roalfe

**Affiliations:** 1 University of Oxford, Oxford, UK

This is a well written protocol for a systematic review of the efficacy and safety of sacubitril/valsartan in the treatment of heart failure. It includes comprehensive search strategies across both published and unpublished sources. The review will provide a valuable appraisal of the evidence to date.

 The protocol could be improved by clarifying in the abstract that the review will consider all subtypes of heart failure. Although the main text states that all subtypes will be included, this is unclear in the abstract.

 The list of outcomes is comprehensive, however, it would be helpful to categorise the outcomes by efficacy and safety.

 Consideration is given for sensitivity and subgroup analyses of published/unpublished and publication/trial registry/CSR, however, the efficacy and safety of treatment within heart failure subtypes should also be considered. How will the reviewers synthesize data where different definitions of HFrEF etc are used?

Is the study design appropriate for the research question?

Yes

Is the rationale for, and objectives of, the study clearly described?

Yes

Are sufficient details of the methods provided to allow replication by others?

Yes

Are the datasets clearly presented in a useable and accessible format?

Not applicable

Reviewer Expertise:

Medical statistics

I confirm that I have read this submission and believe that I have an appropriate level of expertise to confirm that it is of an acceptable scientific standard.

# Response

**Published:** 2021-01-26

**Authors:** David Byrne

**Affiliations:** Royal College of Surgeons in Ireland, Ireland

**The protocol could be improved by clarifying in the abstract that the review will consider all subtypes of heart failure. Although the main text states that all subtypes will be included, this is unclear in the abstract.**

 Response: The abstract was updated to reflect that all categories of heart failure will be included in the analysis: “Heart failure of any subtype or NYHA class will be included”.

**The list of outcomes is comprehensive, however, it would be helpful to categorise the outcomes by efficacy and safety.**

 Response: The outcomes section has been updated to reflect the different categories, namely efficacy and safety outcomes.

**Consideration is given for sensitivity and subgroup analyses of published/unpublished and publication/trial registry/CSR, however, the efficacy and safety of treatment within heart failure subtypes should also be considered. How will the reviewers synthesize data where different definitions of HFrEF etc are used?**

 Response: There are variations in the definitions of HFrEF used in various studies, and this is an important issue to address. The evidence synthesis section has been updated to give further clarification on how this will be addressed in this study: “Analysis will be performed using pooled data of all heart failure subtypes. Where estimates of efficacy and safety are available for individual heart failure subtypes and where consistent definitions of ejection fraction are used across studies, analysis will be performed separately for any subtypes e.g. preserved ejection fraction and reduced ejection fraction.”

# Peer review: Reviewer response for version 1

**DOI:** 10.21956/hrbopenres.14033.r27365

**Published:** 2020-05-15

**Authors:** Li Wei

**Affiliations:** 1 Research Department of Practice and Policy, University College London School of Pharmacy, London, UK

This is a well written protocol on a very important clinical and public health issue. The authors have provided an excellent scientific background about a systemic review of the efficacy and safety of sacubitril/valsartan medications in the treatment of heart failure. The research questions, aims and objectives are very clear. I believe that the results of this review would provide the best scientific evidence to support current clinical practice in using sacubitril/valsartan medications.

 The protocol is structured using the PRISMA-P guidelines and the contents in each section are clear and concise. The outcomes would be clearer if they could be categorised into two groups: efficacy outcomes and safety outcomes as efficacy and safety are two separate concepts.

 Sacubitril/valsartan was approved by both the European Medicines Agency (EMA) and Food and Drug Administration (FDA) in 2015. Therefore database search from inception may not be necessary.

 Risk of bias in individual studies will be conducted independently by two reviewers. It is worth to add an extra sentence that any discrepancy will be resolved through discussion or in consultation with a third researcher.

Is the study design appropriate for the research question?

Yes

Is the rationale for, and objectives of, the study clearly described?

Yes

Are sufficient details of the methods provided to allow replication by others?

Yes

Are the datasets clearly presented in a useable and accessible format?

Not applicable

Reviewer Expertise:

Pharmacoepidemiology and drug safety research

I confirm that I have read this submission and believe that I have an appropriate level of expertise to confirm that it is of an acceptable scientific standard.

# Response

**Published:** 2021-01-26

**Authors:** David Byrne

**Affiliations:** Royal College of Surgeons in Ireland, Ireland

**The outcomes would be clearer if they could be categorised into two groups: efficacy outcomes and safety outcomes as efficacy and safety are two separate concepts.**

 Response: The outcomes section has been updated to reflect the different categories, namely efficacy and safety outcomes.

**Risk of bias in individual studies will be conducted independently by two reviewers. It is worth to add an extra sentence that any discrepancy will be resolved through discussion or in consultation with a third researcher.**

 Response: The risk of bias section has been updated to outline that a third researcher will review and resolve any issues of discrepancy: “This will be conducted independently by two reviewers with any discrepancies or disagreements resolved with the assistance of a third reviewer”.

# Peer review: Reviewer response for version 1

**DOI:** 10.21956/hrbopenres.14033.r27154

**Published:** 2020-05-11

**Authors:** Kamal R. Mahtani

**Affiliations:** 1 Centre for Evidence‐Based Medicine, Nuffield Department of Primary Care Health Sciences, Radcliffe Observatory Quarter, University of Oxford, Oxford, UK

This systematic review protocol seeks to study the efficacy and safety of sacubitril/valsartan medications in the treatment of heart failure. The authors make a clear and relevant rationale for why this systematic review is needed now. The aims and objectives appear clear. 

 The authors are innovatively attempting to obtain clinical study reports from the European Medicines Agency to enhance their data sources. The authors should provide some details on how they plan to manage the potentially large volumes of data that they may receive. For example, how do they plan to index these volumes as well develop a strategy to search and access data within them? 

 Although the authors make some partial reference to this, they may wish to state more explicitly how they plan to compare any differences in the published randomised control trial data versus the unpublished clinical study report data.   

 Overall, I expect this to be a vital review that will add to our knowledge base.

Is the study design appropriate for the research question?

Yes

Is the rationale for, and objectives of, the study clearly described?

Yes

Are sufficient details of the methods provided to allow replication by others?

Yes

Are the datasets clearly presented in a useable and accessible format?

Not applicable

Reviewer Expertise:

Evidence synthesis, drug therapeutics

I confirm that I have read this submission and believe that I have an appropriate level of expertise to confirm that it is of an acceptable scientific standard.

# Response

**Published:** 2021-01-26

**Authors:** David Byrne

**Affiliations:** Royal College of Surgeons in Ireland, Ireland

**Point 1: The authors should provide some details on how they plan to manage the potentially large volumes of data that they may receive. For example, how do they plan to index these volumes as well develop a strategy to search and access data within them?**

 Response: The data management section has been updated to provide further information on the steps that will be followed to index relevant CSR document sections to facilitate searching and extraction of data.

**Point 2: Although the authors make some partial reference to this, they may wish to state more explicitly how they plan to compare any differences in the published randomised control trial data versus the unpublished clinical study report data.**   

 Response: The differences in outcome data between unpublished and published sources is a central focus of this study. We have expanded the data synthesis section to provide more details on dealing with data from various sources, including sensitivity analysis to assess the impact of data source (trial publication, registry, or CSR) on the relevant effect estimates: “Data will be extracted separately from each source, namely trial publication, trial registry and CSR. Both quantitative and qualitative data will be extracted including clinical efficacy and safety outcomes as well as, where relevant, details of trial characteristics and design … Sensitivity analysis will then also be conducted for each outcome to meta-analyse data from each source separately in order to assess the impact on the effect estimate.”
